# Cerebrovascular accidents in paediatric patients supported by the Berlin Heart EXCOR

**DOI:** 10.1093/ejcts/ezac381

**Published:** 2022-07-18

**Authors:** Sofie Rohde, Eugen Sandica, Kevin Veen, Oliver Miera, Antonio Amodeo, Carlo Pace Napoleone, Mustafa Özbaran, Joanna Sliwka, Timothy Thiruchelvam, Daniel Zimpfer, Stephan Schubert, Ad J J C Bogers, Theo M M H de By

**Affiliations:** Department of Cardio-thoracic surgery, Erasmus University Medical Center, Rotterdam, the Netherlands; Clinic for Pediatric Cardiac Surgery and Congenital Heart Defects, Heart and Diabetes Centre North Rhine-Westphalia, Ruhr-University of Bochum, Bad Oeynhausen, Germany; Department of Cardio-thoracic surgery, Erasmus University Medical Center, Rotterdam, the Netherlands; Department of Congenital Heart Disease and Pediatric Cardiology, Deutsches Herzzentrum Berlin, Berlin, Germany; Ospedale Bambino Gesù, Rome, Italy; Pediatric Cardiac Surgery Department, Regina Margherita Children’s Hospital, Torino, Italy; Ege University Hospital, Izmir, Turkey; Department of Cardiac Surgery, Transplantology and Vascular Surgery, Silesian Center for Heart Diseases, Zabrze, Poland; Great Ormond Street Hospital, London, United Kingdom; Vienna Medical University, Vienna, Austria; Center of Congenital Heart Disease/Pediatric Cardiology, Heart and Diabetes Center North Rhine Westfalia, Ruhr-University of Bochum, Bad Oeynhausen, Germany; Department of Cardio-thoracic surgery, Erasmus University Medical Center, Rotterdam, the Netherlands; EUROMACS, EACTS House, Windsor, United Kingdom

**Keywords:** Paediatric, Ventricular assist device, Berlin Heart, Cardiac transplantation, Cerebrovascular accidents

## Abstract

**OBJECTIVES:**

Ventricular assist device support as a bridge to transplant or recovery is a well-established therapy in children on the cardiac transplant waiting list. The goal of this study was to investigate the incidence of and the associated factors for cerebrovascular accidents in paediatric patients supported by a Berlin Heart EXCOR.

**METHODS:**

All patients <19 years of age supported by a Berlin Heart EXCOR between January 2011 and January 2021 from the European Registry for Patients with Mechanical Circulatory Support were included.

**RESULTS:**

In total, 230 patients were included. A total of 140 (60.9%) patients had a diagnosis of dilated cardiomyopathy. 46 patients (20.0%) sustained 55 cerebrovascular accidents, with 70.9% of the episodes within 90 days after the ventricular assist device was implanted. The event rate of cerebrovascular accidents was highest in the first era (0.75). Pump thrombosis and secondary need for a right ventricular assist device were found to be associated with a cerebrovascular accident (hazard ratio 1.998, *P* = 0.040; hazard ratio 11.300, *P* = 0.037). At the 1-year follow-up, 44.4% of the patients had received a transplant, 13.1% were weaned after recovery and 24.5% had died. Event rates for mortality showed a significantly decreasing trend.

**CONCLUSIONS:**

Paediatric ventricular assist device support is associated with important adverse events, especially in the early phase after the device is implanted. Pump thrombosis and the need for a secondary right ventricular assist device are associated with cerebrovascular accidents. Furthermore, an encouragingly high rate of recovery in this patient population was shown, and death rates declined. More complete input of data into the registry, especially concerning anticoagulation protocols, would improve the data.

**Figure:**
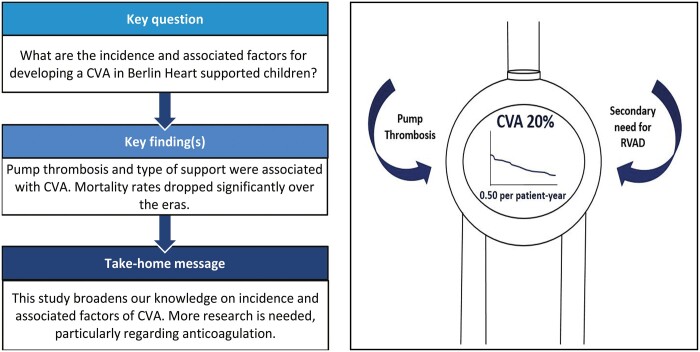

## INTRODUCTION

Ventricular assist devices (VAD) have become an important aspect in the treatment of children with end-stage heart failure and often act as a last resort for these severely sick children awaiting a cardiac transplant. They have proven to lower waiting list mortality [1] and do not compromise post-transplant outcomes [2]. VAD therapy can also be used as a bridge to a decision or as a bridge to recovery. The majority of children supported by a VAD are diagnosed with cardiomyopathy; however, VAD support can also be a viable option for children with end stage congenital heart disease (CHD) [2, 3]. Many of the patients have a high Interagency Registry for Mechanically Assisted Circulatory Support (INTERMACS) patient profile and are on inotropes, reflecting the severity of their clinical condition [3].

In younger children, the Berlin Heart EXCOR (BHE) (Berlin Heart GmbH, Berlin, Germany) is one of the most used VADs for long-term support. It is designed to provide long-term support and obtained U.S. Food and Drug Administration approval in 2008. Because of the paracorporeal position of this device, smaller patients can be treated as well. Different pump sizes are available to match the size of the patient.

Support with a BHE, however, comes with a certain incidence of adverse events. Cerebrovascular accidents (CVA) are of particular concern. In adults, the incidence of CVA is estimated at around 13% at 1 year after the VAD is implanted [5]. In children supported by a BHE, the incidence is higher and has been reported to be up to 50% [6–8]. Other frequently reported adverse events are bleeding, infection and pump thrombosis [3, 4].

Despite the continued increase in usage of VAD therapy in paediatric patients, the single-centre experiences remain low. Joint efforts such as The European Registry for Paediatric Patients with Mechanical Circulatory Support (Paedi-EUROMACS) are needed to better understand the clinical course of these severely ill children and how to attain the best pre- and post-transplant outcomes. From the analyses of this registry, lessons can be learned to improve clinical care for VAD-supported patients. Because CVA is one of the principal complications in the clinical course of paediatric patients supported by a BHE, the goal of this study was to elucidate the incidence rates of and the associated factors for CVA in the population of patients with the BHE.

## PATIENTS AND METHODS

For this analysis, we included all paediatric patients (<19 years old) from the EUROMACS database supported by a BHE between 2011 and January 2021. The age threshold was chosen in accordance with the first 2 Paedi-EUROMACS reports [3, 9]. Twenty-five centres are included in this database. The progress and the input of every site are monitored once every 2 months per site. We identified 2 groups within this population: patients who had a CVA after a VAD was implanted and a non-CVA group. For additional analyses we divided our population into 3 eras: era I: January 2011 through April 2014; era II: May 2014 through August 2017; era III: September 2017 through December 2020. Secondary outcomes were other major adverse events, death and a transplant. CVA and adverse events are reported according to the INTERMACS definitions [10]. CVA included transient ischaemic attacks, ischaemic stroke or intracranial haemorrhage diagnosed either by clinical acute (transient) neurological deficit conforming anatomically to arterial distribution cerebral ischaemia or by brain imaging. Early adverse events were defined as occurring within 30 days after the VAD was implanted. Most centres use the Edmonton protocol as the guideline for their antithrombotic strategy [11]. However, many centres deviate significantly from the original recommendations [12]. Precise information on which anticoagulation strategy was used is not available in the EUROMACS database. A table of missing data is presented in the supplemental material.

### Ethics statement

All individual hospitals received approval from their MEC/research ethics committee in accordance with the policy of the *European Journal of Cardio-Thoracic Surgery*.

### Statistical analyses

Continuous data are presented as median [interquartile range (IQR)] and categorical data are presented as frequencies (percentage). The Student *t*-test or the Mann–Whitney test was used to compare continuous variables. The χ^2^ test or the Fisher exact test was used to compare categorical variables. Because CVA is a competing event associated with weaning, a transplant and death, the cumulative incidence of CVA is estimated using Fine and Gray models.

### Associated factors

To analyse which covariates are associated with CVA, univariable Cox proportional hazard regression models were developed. Proportional hazard assumptions were tested using Schoenfeld residuals. Infection, pump thrombosis and major bleeding are time-dependent covariates that occur during the follow-up period. To account for these phenomena, the Cox proportional hazard model was extended using time-varying covariates. Furthermore, post hoc trend analyses were performed. Missing data were imputed using multiple imputation by chained equations using all baseline data. In total, 20 imputed data sets were creating using 40 iterations each. Convergence was checked using convergence plots, and imputations were visually checked using density plots.

### Statistical program

All analyses were performed using International Business Machines Corporation Statistical Package for the Social Sciences (IBM SPSS, Armonk, NY, USA) statistics (version 24) or R (Version 4.0.3) with the packages “Survival”, “cmprsk” and “gpreg” (R Foundation for Statistical Computing, Institute for Statistics and Mathematics, Vienna, Austria).

## RESULTS

A total of 230 patients under 19 years of age supported by a BHE were included. Of these, 48.3% were male, and the median age was 2.0 years (IQR 0.6–8.0). A total of 31.7% of the patients were below 1 year of age, and only 14.3% were older than 10 years of age. The most common primary diagnosis was dilated cardiomyopathy (60.9%), but the aetiology differed by era (*P* = 0.002) (Table 1; Table 5, supplemental material).

**Table 1:** Perioperative characteristics of patients with and without cardiovascular accidents

	All	CVA	Non-CVA
	(n = 230)	(n = 46)	(n = 184)
Male sex, n (%)	111 (48.3)	20 (43.5)	91 (49.5)
Age (years), median (IQR)	2.0 (0.6-8.0)	1.0 (0.5-4.3)	3.0 (0.8-8.0)
BSA (m^2^), median (IQR)	0.5 (0.4-0.9)	0.5 (0.4-0.7)	0.6 (0.4-0.9)
Primary diagnosis			
CHD, n (%)	49 (21.3)	8 (17.4)	41 (22.3)
DCM, n (%)	140 (60.9)	32 (69.6)	108 (58.7)
RCM, n (%)	18 (7.8)	4 (8.7)	14 (7.6)
Other^b^, n (%)	5 (2.2)	0	5 (2.7)
Unknown, n (%)	18 (7.8)	2 (4.3)	16 (8.7)
Time since first cardiac diagnosis			
Less than 1 month, n (%)	81 (35.2)	16 (34.8)	65 (35.3)
One month to 1 year, n (%)	52 (22.6)	13 (28.3)	39 (21.2)
One to 2 years, n (%)	22 (9.6)	8 (17.4)	14 (7.6)
Over 2 years, n (%)	50 (21.7)	7 (15.2)	43 (23.4)
Unknown, n (%)	25 (10.9)	2 (4.3)	23 (12.5)
Creatinine, median (IQR)	44.0 (33.0-61.0)	42.0 (26.5-61.0)	45.0 (34.0-61.0)
Albumin, median (IQR)	528.9 (409.3-659.7)	543.4 (434.7-668.0)	514.4 (387.6-659.3)
NT-pro-BNP, median (IQR)	20528.0 (8592.0-35000.00)	28518.5 (10060.0-35302.5)	17764.0 (8575.5-35000.0)
INTERMACS classification			
I, n (%)	53 (23.0)	11 (23.9)	42 (22.8)
II, n (%)	113 (49.1)	24 (52.2)	89 (48.4)
III-V, n (%)	45 (19.6)	9 (19.6)	36 (19.6)
Unknown, n (%)	19 (8.3)	2 (4.3)	17 (9.2)
Atrial fibrillation or atrial flutter, n (%)	7 (3.0)	1 (2.2)	6 (3.3)
Previous intubation, n (%)	81 (38.8)	15 (33.3)	66 (40.2)
Previous dialysis, n (%)	7 (3.3)	1 (2.2)	6 (3.7)
Previous ECMO, n (%)	44 (21.5)	8 (17.8)	36 (22.5)
Previous cardiac surgery, n (%)	37 (16.1)	9 (20.0)	28 (17.4)
Previous cardiac arrest, n (%)	29 (13.9)	7 (15.6)	22 (13.4)
Device strategy			
(Possible) bridge to transplant, n (%)	191 (83.0)	40 (87.0)	151 (82.1)
Bridge to recovery, n (%)	20 (8.7)	5 (10.9)	15 (8.2)
Unknown, n (%)	19 (8.3)	1 (2.2)	18 (9.8)
Type of support			
LVAD, n (%)	168 (73.0)	35 (76.1)	133 (72.3)
BiVAD, n (%)	54 (23.5)	10 (21.7)	44 (23.9)
LVAD + RVAD^c^, n (%)	7 (3.0)	1 (2.2)	6 (3.3)
RVAD, n (%)	1 (0.4)	0	1 (0.5)
Duration of support (days), median (IQR)	143 (39-249)	89 (39-222)	184 (30-237)
Patients with early pump thrombosis, n (%)	31 (13.5)	14 (30.4)	18 (9.8)
Total episodes of early pump thrombosis	50	23	28
Patients with early major bleeding, n (%)	26 (11.3)	5 (10.9)	21 (11.4)
Total episodes of early major bleeding	37	8	29
Patients with early major infection, n (%)	22 (9.6)	5 (10.9)	17 (9.2)
Total episodes of early major infection	23	5	18

a This table is not by any means to represent any possible association between covariates and the risk of CVA, merely to give an idea of the composition of the 2 groups.

b Other: valvular heart disease in 3 patients, hypertrophic cardiomyopathy in 1 patient and cancer in 1 patient.

c When the RVAD is placed during a second operation.

BiVAD: biventricular assist device; BSA: body surface area; CHD: congenital heart disease; CVA: cerebrovascular accident; DCM: dilated cardiomyopathy; ECMO: extracorporeal membrane oxygenation; INTERMACS: Interagency Registry for Mechanically Assisted Circulatory Support; LVAD: left ventricular assist device; RCM: restrictive cardiomyopathy; RVAD: right ventricular assist device.

**Figure 1:**
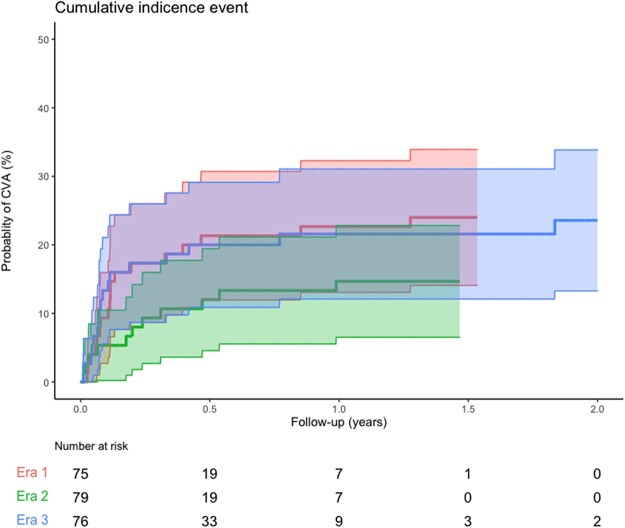
Cumulative incidence function plot of cardiovascular accidents stratified by era. Era I: January 2011 through April 2014; era II: May 2014 through August 2017; era III: September 2017 until January 2021. CVA: cerebrovascular accident.

In total, 46 patients sustained 55 CVAs. Twenty-one episodes (38.2%) occurred in the first 30 days after the VAD was implanted, and 39 events (70.9%) occurred in the first 90 days (Table 1) (Fig. 1). The cumulative incidence of CVA did not significantly differ per era, and there was no significant trend (*P* = 0.458) (Fig. 1; Table 2). Twenty-five of the 55 (45.5%) CVA episodes contributed to the death of a patient, with a total event rate of 0.23 per patient-year (Table 2; Table 5, supplemental material).

**Table 2:** Event rates of primary and secondary outcomes per era per patient-year

	All	Era I	Era II	Era III	*P-*_trend_
	(n = 230)	(n = 75)	(n = 79)	(n = 76)	
CVA	0.50	0.75	0.36	0.44	0.458
iCVA	0.13	0.25	0.11	0.05	^a^
hCVA	0.17	0.19	0.17	0.17	^a^
uCVA	0.20	0.31	0.08	0.22	^a^
Contributed to death	0.23	0.28	0.14	0.27	0.959
Pump thrombosis	1.91	2.11	2.08	1.62	0.299
Major bleeding	0.45	0.41	0.36	0.56	0.488
Major infection	0.47	0.35	0.33	0.68	0.411
Died	0.52	0.63	0.53	0.44	0.019
Transplant	1.15	1.01	1.25	0.92	0.830
Recovery	0.29	0.38	0.28	0.24	0.154
Ongoing	0.09		0.03	0.22	0.253
Lost to follow-up	0.05		0.11	0.02	0.891

a Because many episodes of cardiovascular accidents were not specified as either ischaemic or haemorrhagic, we did not perform these analyses.

CVA: cardiovascular accident; hCVA: haemorrhagic CVA; iCVA: ischaemic CVA; uCVA: unspecified CVA.

With a univariable Cox proportional hazard regression model, our goal was to identify associated factors for developing a CVA. Pump thrombosis was found to be associated with early and late CVA combined [hazard ratio (HR) 1.998, *P* = 0.040]. Additionally, the secondary implant of an RVAD after an LVAD was significantly associated with an increased risk of CVA compared to an LVAD only (HR 11.300, *P* = 0.037). However, the large HR and extremely wide confidence intervals indicated that there are limited data to support a robust conclusion.

Secondary outcomes were other major adverse events, death and a transplant. Pump thrombosis had a total event rate of 1.91 per patient-year, with the highest event rate in the first and second eras (2.11 and 2.08 per patient-year, *P* = 0.299). In the third era, a remarkably low percentage of patients experienced an early pump thrombosis (5.3% compared to 17.3–19.0, *P* = 0.028). Pump thrombosis was associated with CVA (HR 1.998, *P* = 0.040). A total of 75.5% of all the major bleeds and 45.1% of the major infections occurred within 30 days after the VAD was implanted. A list of specified infections reported in our population can be found in Table 4 (supplemental material). Major infection or major bleeding was not associated with CVA (Tables 1–3) (Table 5, supplemental material).

**Table 3:** Univariable Cox proportional hazard regression model: associated factors for developing a cardiovascular accident

	Hazard ratio	95% CI	*P*-value
Era: II _versus era I_	0.566	0.267-1.200	0.138
Era: III _versus era I_	0.731	0.372-1.437	0.364
Male sex	0.899	0.488-1.654	0.726
Age	1.046	0.976-1.122	0.198
BSA	1.756	0.754-4.089	0.186
Primary diagnosis: DCM _versus CHD_	1.883	0.787-4.504	0.150
Primary diagnosis: RCM _versus CHD_	3.262	0.872-12.206	0.078
Creatinine	0.997	0.984-1.009	0.588
Albumin	1.000	1.000-1.000	0.447
NT-pro-BNP	1.000	1.000-1.000	0.624
INTERMACS: II _versus INTERMACS I_	1.011	0.486-2.102	0.976
INTERMACS: III-V _versus INTERMACS I_	2.562	0.999-6.569	0.050
Previous intubation	1.662	0.866-3.189	0.123
Previous ECMO	1.061	0.468-2.408	0.885
Previous cardiac surgery	0.718	0.331-1.558	0.394
Previous cardiac arrest	1.161	0.510-2.642	0.716
Device strategy: Bridge to recovery _versus bridge to transplant_	1.258	0.491-3.225	0.625
Type of support: BiVAD _versus LVAD_	1.238	0.592-2.589	0.562
Type of support: LVAD + RVAD _versus LVAD_	11.300	1.172-108.927	0.037
Major infection^a^	1.104	1.420-32.298	0.866
Cumulative major Infection	0.254	1.044-4.563	0.133
Pump thrombosis^a^	1.998	1.031-3.872	0.040
Major bleeding^a^	1.104	0.450-2.707	0.829

a Time varying covariate.

BiVAD: biventricular assist device; BSA: body surface area; CVA: cerebrovascular accident; ECMO: extracorporeal membrane oxygenation; fDCM: familial dilated cardiomyopathy; iDCM: idiopathic dilated cardiomyopathy; INTERMACS: Interagency Registry for Mechanically Assisted Circulatory Support; LVAD: left ventricular assist device; mDCM: myocarditis dilated cardiomyopathy; oDCM: other dilated cardiomyopathy; RCM: restrictive cardiomyopathy; RVAD: right ventricular assist device.

**Figure 2:**
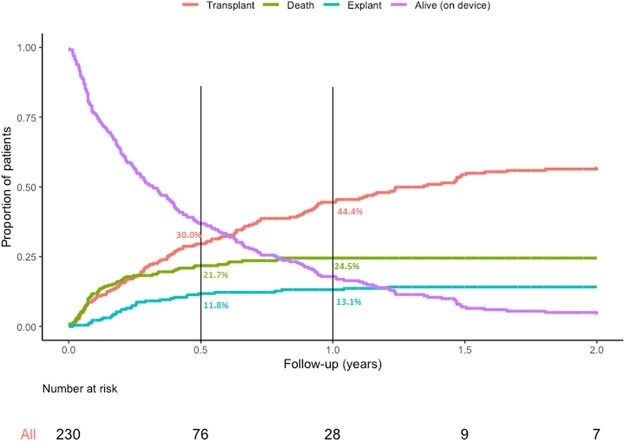
Competing outcomes.

In total, 126 patients (56.0%) survived to transplant with a 6-month transplant rate of 30.0% and a 1-year transplant rate of 44.4%. The overall event rate for a transplant was 1.15 per patient-year. In 32 children (14.2%), the BHE could be explanted due to recovery, with most explants occurring in the first half-year after the VAD was implanted (84.4%). The overall event rate for an explant due to recovery was 0.29 per patient-year. Overall mortality was 25.3% (57 patients) with a 6-month mortality of 21.7% and a 1-year mortality of 24.5%. The event rate for mortality decreased significantly over the eras (*P* = 0.019) (Table 2; Fig. 2).

## DISCUSSION

In our population, 46 (20.0%) patients suffered from a CVA, with 55 events in total (event rate 0.50 per patient-year) (Table 2). Our North American colleagues reported a comparable incidence of 16.9% CVAs in patients supported by a pulsatile paracorporeal device [13]. However, their reported event rate of 6.4 per 100 patient months, which equals an event rate of 0.77 per patient-year, was higher [13]. This result could be partially explained by the higher overall rates of paediatric transplants at 1 year (62.4% in the United States vs 46.7% in Europe) and therefore a shorter median time on support, while most of the CVAs occur in the first months of VAD support [3, 4]. In single-centre cohort studies, CVA rates differ widely from 25.0 to 50.0% [6–8, 14]. These differences can be explained by multiple factors, for example, differences in follow-up time and patient characteristics.

### Associated factors

Because the incidence of CVA is related to VAD therapy and the concomitant need for anticoagulation, most CVAs are reported in the early support period [8, 13, 15, 16]. In the first week of VAD support, anticoagulation therapy must be carefully initiated. Overdosing anticoagulation shortly after the operation can cause bleeding requiring reoperation, but a too small dose exposes the patient to a higher risk of thromboembolic events. Therefore, most bleeding and thromboembolic events occur in this period when this delicate balance is being achieved [15, 17, 18]. Surprisingly, our analyses showed only 38.2% of the CVAs occurring in the first 30 days after the VAD is implanted. However, 70.9% occurred in the first 90 days after the VAD was implanted. Furthermore, 37 of the 49 major bleeding events (75.5%) occurred in the first 30 days of VAD support. The Edmonton protocol is historically the most used anticoagulation protocol in paediatric VAD-supported patients [11]. This protocol has of yet not been thoroughly analysed, because clinicians often deviate from it, resulting in wide intercentre variability [12, 19]. Unfortunately, the EUROMACS database does not contain information on the precise anticoagulation strategy used. More research is warranted to optimize anticoagulation therapy in children supported by a BHE.

Another associated factor for CVA found in this study is the need for a secondary RVAD (HR 11.300, *P* = 0.037) compared to univentricular support alone. However, biventricular support from the beginning—that is, if the LVAD and RVAD were placed during the first operation—versus univentricular support alone did not reach statistical significance. This finding is interesting, since to our knowledge, this has not been explored in great detail by other major studies. It may reflect a clinical deterioration (as there was initially no need to support both ventricles) accompanied by a disturbance in the delicate balance between coagulation and anticoagulation, during which a higher risk for CVA appears to emerge. Although the placement of an secondary RVAD compared to an univentricular support alone was significantly associated with CVA, this result should be interpreted with caution because the confidence interval is wide and the number of patients supported by the placement of a second RVAD is low.

Additionally, other investigators found a later era to be a protective factor against CVA in the children supported with a paracorporeal pulsatile VAD (HR 0.15, 95% CI 0.05–0.45, *P* = 0.0006), which could indicate a learning curve of the participating centres [13]. In our population, event rates for CVA were highest in era I, but the trend was not significant (*P* = 0.458) (Table 2, and support in a later era was not proven to be a protective factor against developing a CVA (*P* = 0.129, *P* = 0.364) (Table 3).

Another variable often reported as a risk factor for a worse outcome is CHD as an initial diagnosis compared to other aetiologies such as cardiomyopathies [4, 20]. For example, survival at 12 months after a VAD implant was significantly lower in the CHD group compared to the cardiomyopathy group in the analysis of the North American registry (60.9% vs 76.8%, *P* < 0.0001) [4], and CHD was an independent predictor of waiting list mortality (OR 2.4, 95% CI 1.9–3.0) . In this study, cardiomyopathy as the primary diagnosis was not a significant predictive factor (HR 1.883, *P* = 0.150; HR 3.262, *P* = 0.078) (Table 3). Our North American colleagues also did not find an association between CHD and CVA in VAD-supported children [13]. CHD appears, therefore, to be a predictive factor for overall mortality but not for CVA.

Lastly, younger age has been reported to predict higher mortality and lower transplant rates in VAD-supported children [4]. In small single-centre studies, incidence rates of CVA between 14% and 38% have been reported in patients under 1 year of age supported by a BHE [21–23]. In our population, 31.7% of the patients were below 1 year of age and only 14.3% were above 10 years of age. Age was not a predictive factor for developing a CVA (HR 1.046, *P* = 0.198) (Table 3).

### Relationship to comorbidities

Pump thrombosis was found to be associated with CVA (HR 1.998, *P* = 0.040), in accordance with previously published results [18]. The relationship between infection and CVA was also considered. In total, 51 infections occurred in 34 patients, with 45.1% occurring within 30 days after a VAD was implanted. Similarly, in the Pedimacs registry, 17% of the reported adverse events were infections, which occurred mostly in the first 30 days after the VAD was implanted. A history of prior non-infectious major adverse events such as neurological dysfunction was associated with infection (HR 1.9, 95% CI 1.0–3.8, *P* = 0.05) [24]. This interesting association between infectious complications and CVA is to be expected, because CVA prolongs the hospital stay and therefore the risk of hospital-acquired infections. Furthermore, inflammation can cause hypercoagulation by influencing certain clotting factors and, likewise, coagulation products can stimulate an inflammatory response [25]. Byrnes *et al.* [17], therefore, reported administering steroids to patients with a systemic inflammatory response syndrome, without a positive blood culture and with a fibrinogen blood level >600 mg/dl. This protocol may have contributed to the decreased incidence of CVAs. However, our study did not show an association between (recurrent) infections and the occurrence of CVA. Furthermore, major bleeding was, surprisingly, also not associated with CVA, even though one might expect that a patient at risk for a major bleeding event elsewhere in the body is probably also more at risk for a haemorrhagic CVA. Moreover, a patient with major bleeding might receive less anticoagulants after the event, increasing the risk of an ischaemic CVA.

### Final outcomes

In our population, 44.4% of the patients survived to transplant. In 11.8%, the BHE could be explanted due to myocardial recovery at 1 year of follow-up. Our colleagues report a higher transplant rate at 1 year in patients supported with a paracorporeal pulsatile device of 74.4%, but a lower 1-year recovery rate of 4.7% [4]. The trend, however, is comparable: Almost all episodes of VAD explants happened in the first 6 months, whereas around 15% of the transplants in both populations took place between 0.5 and 1.0 years [4]. Additionally, the event rate for mortality was highest in era I (0.63 per patient-year) and declined over the years that followed (*P* = 0.019), suggesting a possible learning curve of the health care provided to these children. A certain learning curve has been suggested in the literature [8].

### Limitations

This study has some limitations. Firstly, this study was limited by missing data, including a lack of detailed information on the type of CVA (i.e. ischaemic or haemorrhagic), the severity and duration of the symptoms and information regarding the antithrombotic therapy used, which would have been of great value. To minimize the impact of missing data in the statistical analyses, we used multiple imputation with chained equations. Secondly, since this is a registry-based study, we received information on the included patients from various centres from different people. Detection bias might have influenced the data we used. We made an effort to minimalize this by providing extensive adverse event definitions to every participating centre. Lastly, despite only considering children with a BHE, significant interpopulation variance was still observed. For example, we included patients from 0 to 18 years old with INTERMACS patient profiles from I to V. It is clear that despite selecting for this narrow population, several aspects of their clinical courses differ widely, including aspects that could potentially influence CVA rates (e.g. organ availability, therapy compliance, mortality, morbidity).

## CONCLUSION

This study is of great importance because it covers the complete EUROMACS experience of children supported with a BHE. This study shows that paediatric VAD support is associated with adverse events, including CVAs, most of which occur in the early support period. Pump thrombosis and type of support were found to be associated with CVA. Surprisingly, (recurrent) infections and major bleeding as potential factors associated with CVA did not achieve statistical significance. At the 1- year follow-up, more than half of our included patients had undergone a transplant or had recovered. Ensuring future comprehensive data entry into the registry will optimize research output, which in turn will provide a basis on which further research and subgroup analysis in the paediatric VAD population can be performed. In particular, strategies for anticoagulation therapy should be studied further.

## FUNDING

The European Association for Cardio-Thoracic Surgery supported this study and made resources available to execute this work. No funding was received for this work.

**Conflict of interest:** None of the authors have conflicts of interest to disclose.

## Supplementary Material

ezac381_Supplementary_Data

## Data Availability

All relevant data are within the manuscript and its Supporting Information files.
