# Supplementary material for: Cerebrovascular accidents in paediatric patients supported by the Berlin Heart EXCOR
Source: Eur J Cardiothorac Surg. 2022 Jul 18;62(3):ezac381. doi: 10.1093/ejcts/ezac381 (PMC9789740; doi:10.1093/ejcts/ezac381)
Supplement: ezac381_Supplementary_Data [file ezac381_supplementary_data.docx]

**Supplemental material**

**Table 4:** Description of different infections reported

| **Major infections specified** | **n** |
| --- | --- |
| Positive blood cultures | 12 |
| Pump related | 9 |
| Pulmonary | 9 |
| Sepsis | 4 |
| Mediastinum | 3 |
| Urinary tract | 3 |
| Gastro-intestinal | 2 |
| Line sepsis | 2 |
| Peripheral wound | 1 |
| Other | 1 |
| Unknown | 5 |

**Table 5**: Perioperative characteristics stratified by Era

|  | **All (n=230)** | **Era I (n=75)** | **Era II (n=79)** | **Era III (n=76)** | **P** |
| --- | --- | --- | --- | --- | --- |
| Male sex, n (%) | 111 (48.3) | 38 (50.7) | 40 (50.6) | 33 (43.4) | 0.587 |
| Age (years), median (IQR) | 2.0 (0.6-8.0) | 2.0 (0.7-7.0) | 3.0 (0.3-7.0) | 2.0 (0.8-8.0) |  |
| BSA (m^2^), median (IQR) | 0.5 (0.4-0.9) | 0.5 (0.4-0.8) | 0.5 (0.3-0.9) | 0.6 (0.4-0.9) | 0.578 |
| Primary diagnosis |  |  |  |  | 0.002 |
| CHD, n (%) | 49 (21.3) | 25 (33.3) | 18 (22.8) | 6 (7.9) |  |
| DCM, n (%) | 140 (60.9) | 38 (50.7) | 49 (62.0) | 53 (69.7) |  |
| RCM, n (%) | 18 (7.8) | 4 (5.3) | 4 (5.1) | 10 (13.2) |  |
| Other**, n (%) | 5 (2.2) | 0 | 2 (2.5) | 3 (3.9) |  |
| Unknown, n (%) | 18 (7.8) | 8 (10.7) | 6 (7.6) | 4 (5.3) |  |
| Time since first cardiac diagnosis |  |  |  |  | 0.347 |
| Less than one month, n (%) | 81 (35.2) | 24 (32.0) | 29 (36.7) | 28 (36.8) |  |
| One month to a year, n (%) | 52 (22.6) | 20 (26.7) | 17 (21.5) | 15 (19.7) |  |
| One to two years, n (%) | 22 (9.6) | 3 (4.0) | 7 (8.9) | 12 (15.8) |  |
| Over two years, n (%) | 50 (21.7) | 18 (24.0) | 16 (20.3) | 16 (21.1) |  |
| Unknown, n (%) | 25 (10.9) | 10 (13.3) | 10 (12.7) | 5 (6.6) |  |
| Creatinine, median (IQR) | 44.0 (33.0-61.0) | 46.0 (35.0-59.5) | 44.5 (37.0-68.3) | 42.0 (29.0-60.0) | 0.162 |
| Albumin, median (IQR) | 528.9 (409.3-659.7) | 507.2 (362.2-590.5) | 486.9 (420.2-633.9) | 608.6 (438.3-724.5) | 0.345 |
| NT-pro-BNP, median (IQR) | 20528.0 (8592.0-35000.00) | 31017.0 (10314.5-38316.0) | 17408.5 (9208.5-35302.5) | 19839 (7601.5-35000.0) | 0.401 |
| INTERMACS classification |  |  |  |  | 0.422 |
| I, n (%) | 53 (23.0) | 18 (24.0) | 21 (26.6) | 14 (18.4) |  |
| II, n (%) | 113 (49.1) | 33 (44.0) | 36 (45.6) | 44 (57.9) |  |
| III-V, n (%) | 45 (19.6) | 17 (22.7) | 16 (20.3) | 12 (15.8) |  |
| Unknown, n (%) | 19 (8.3) | 7 (9.3) | 6 (7.6) | 6 (7.9) |  |
| Atrial fibrillation or atrial flutter, n (%) | 7 (3.0) | 1 (1.3) | 4 (5.1) | 2 (2.6) | 0.382 |
| Previous intubation, n (%) | 81 (38.8) | 23 (30.7) | 29 (36.7) | 29 (38.2) | 0.583 |
| Previous dialysis, n (%) | 7 (3.3) | 2 (2.7) | 2 (2.5) | 3 (3.9) | 0.893 |
| Previous ECMO, n (%) | 44 (21.5) | 10 (13.3) | 14 (17.7) | 20 (26.3) | 0.114 |
| Previous cardiac surgery, n (%) | 37 (18.0) | 9 (12.0) | 15 (19.0) | 13 (17.1) | 0.420 |
| Previous cardiac arrest, n (%) | 29 (13.9) | 2 (2.7) | 16 (20.3) | 11 (14.5) | 0.003 |
| Device strategy |  |  |  |  | 0.984 |
| (Possible) bridge to transplant, n (%) | 191 (83.0) | 61 (81.3) | 65 (82.3) | 65 (85.5) |  |
| Bridge to recovery, n (%) | 20 (8.7) | 6 (8.0) | 7 (8.9) | 7 (9.2) |  |
| Unknown, n (%) | 19 (8.3) | 8 (10.7) | 7 (8.9) | 4 (5.3) |  |
| Type of support |  |  |  |  | 0.544 |
| LVAD, n (%) | 168 (73.0) | 52 (69.3) | 57 (72.2) | 59 (77.6) |  |
| BiVAD, n (%) | 54 (23.5) | 21 (28.0) | 19 (24.1) | 14 (18.4) |  |
| LVAD + RVAD***, n (%) | 7 (3.0) | 1 (1.3) | 3 (3.8) | 3 (3.9) |  |
| RVAD, n (%) | 1 (0.4) | 1 (1.3) | 0 |  |  |
| Duration of support (days), median (IQR) | 143 (39.0-249.0) | 88.0 (31.0-214.0) | 97.0 (26.0-201.0) | 157.0 (40.0-285.5) | 0.382 |
| Patients with early CVA, n (%) | 20 (8.7) | 7 (9.3) | 4 (5.1) | 9 (11.8) | 0.317 |
| Total episodes of early CVA | 21 | 7 | 5 | 9 |  |
| Symptoms persist <24h e | 2 | 1 | 1 | 0 |  |
| Symptoms persist >24h e | 11 | 5 | 2 | 4 |  |
| Ischemic CVA e | 6 | 4 | 1 | 1 |  |
| Haemorrhagic CVA e | 7 | 1 | 4 | 3 |  |
| Contributed to death n (%) | 7 (35.0) | 0 | 0 | 7 (77.8) |  |
| Patients with early pump thrombosis, n (%) | 32 (13.9) | 13 (17.3) | 15 (19.0) | 4 (5.3) | 0.028 |
| Total episodes of early pump thrombosis | 51 | 26 | 20 | 5 |  |
| Patients with early major bleeding, n (%) | 26 (11.3) | 7 (9.3) | 8 (10.1) | 11 (14.5) | 0.560 |
| Total episodes of early major bleeding | 37 | 10 | 12 | 15 |  |
| Patients with early major infection, n (%) | 22 (9.6) | 9 (12.0) | 8 (10.1) | 5 (6.6) | 0.515 |
| Total episodes of early major infection | 23 | 9 | 8 | 6 |  |

*The unknown-group was not included in the analyses.

**Other = valvular heart disease in 3 patients, hypertrophic cardiomyopathy in 1 patient and cancer in 1 patient.

***When the RVAD is placed in a second surgery.

Era I: January 2011 until May 2014, era II: May 2014 until September 2017, era III: September 2017 until January 2021.

Percentages reflect the percentage of patients in which the parameter of concern was present. For the early CVAs, pump thrombosis, major bleedings, and major infections, the number of patients was compared (not the number of episodes).

BiVAD = Biventricular assist device, BSA = body surface area, CHD = congenital heart disease, CVA = cerebrovascular accident, DCM = dilated cardiomyopathy, ECMO = extracorporeal membrane oxygenation, INTERMACS = Interagency Registry for Mechanically Assisted Circulatory Support, LVAD = left ventricular assist device, RCM = restrictive cardiomyopathy, RVAD = right ventricular assist device

**Table 6:** Missing data

|  | **All (n=230)** | **Era I (n=75)** | **Era II (n=79)** | **Era III (n=76)** |
| --- | --- | --- | --- | --- |
| BSA, n (%) | 6 (2.6) | 1 (1.3) | 1 (1.3) | 4 (5.3) |
| Time since first cardiac diagnosis, n (%) | 25 (10.9) | 10 (13.3) | 10 (12.7) | 5 (6.6) |
| Creatinine, n (%) | 79 (34.3) | 33 (44.0) | 29 (36.7) | 17 (22.4) |
| Albumin, n (%) | 122 (53.0) | 51 (68.0) | 43 (54.4) | 28 (36.8) |
| NT-pro-BNP, n (%) | 143 (62.2) | 58 (77.3) | 49 (62.0) | 36 (47.4) |
| INTERMACS classification, n (%) | 19 (8.3) | 7 (9.3) | 6 (7.6) | 6 (7.9) |
| Rhythm, n (%) | 33 (27.4) | 26 (34.7) | 23 (29.1) | 14 (18.4) |
| Device strategy, n (%) | 16 (7.0) | 8 (10.7) | 7 (8.9) | 4 (5.3) |
| Previous intubation, n (%) | 21 (9.1) | 7 (9.3) | 10 (12.7) | 4 (5.3) |
| Previous dialysis, n (%) | 21 (9.1) | 7 (9.3) | 10 (12.7) | 4 (5.3) |
| Previous ECMO, n (%) | 25 (10.9) | 8 (10.7) | 9 (11.4) | 8 (10.5) |
| Previous cardiac surgery, n (%) | 24 (10.4) | 7 (9.3) | 10 (12.7) | 7 (9.2) |
| Cardiac arrest, n (%) | 21 (9.1) | 7 (9.3) | 10 (12.7) | 4 (5.3) |

Era I: January 2011 until May 2014, era II: May 2014 until September 2017, era III: September 2017 until January 2021.

ECMO = extracorporeal membrane oxygenation, INTERMACS = Interagency Registry for Mechanically Assisted Circulatory Support
